# Ultrasonic optic nerve sheath diameter could improve the prognosis of acute ischemic stroke in the intensive care unit

**DOI:** 10.3389/fphar.2022.1077131

**Published:** 2022-12-23

**Authors:** Cong Li, Cui-Cui Wang, Yan Meng, Jia-Yu Fan, Jie Zhang, Li-Juan Wang

**Affiliations:** ^1^ Department of Neurology, The Neuroscience Center, The First Hospital of Jilin University, Jilin University, Changchun, China; ^2^ Department of Rehabilitation, Shaanxi Provincial People’s Hospital, Xi’an, China

**Keywords:** intracranial pressure, stroke, prognosis, non-invasive, optic nerve sheath diameter, ultrasonic measurement

## Abstract

**Objectives:** Stroke patients with high intracranial pressure (ICP) may have poor prognosis. Non-invasive ultrasonic optic nerve sheath diameter (ONSD) could evaluate increased ICP. To investigate whether ONSD is valuable for prognosis of patients with acute ischemic stroke (AIS).

**Methods:** AIS receiving intensive care were recruited with the Glasgow Coma Scale (GCS) score. Patients in group A underwent ultrasonic ONSD to assess ICP voluntarily, whereas group B without ONSD. Patients were followed up at discharge and once a week for 3 months with Glasgow Outcome Scale (GOS) score (four to five scores indicated good prognosis and one to three scores indicated poor prognosis).

**Results:** Forty-nine patients were included. GCS scores did not differ significantly between groups A (26 patients) and B (8 ± 3 vs. 7 ± 3, *p* < 0.05). In group A, ONSD was 5.01 ± 0.48 mm, which correlated with GCS score (*p* < 0.05). At discharge, the GOS score was higher in group A than in group B (3.35 ± 1.35 vs. 2.57 ± 1.121, *p* = 0.034). The proportion of patients with a good prognosis was higher in group A than in group B (46.2% vs. 13.0%, *p* = 0.006). At discharge and after 3 months of follow-up, ONSD at admission was correlated with the GOS score in group A (r = -0.648 [*p* < 0.05] and -0.731 [*p* < 0.05], respectively). After 3 months of follow-up, the GOS score was higher in group A than group B (3.00 ± 1.673 vs. 2.04 ± 1.430, *p* < 0.05). The proportion of patients with a good prognosis was higher in group A than in group B (46.2% vs. 21.2%, *p* = 0.039). The Kaplan-Meier curve showed a higher rate of good prognosis in group A than in group B. ONSD (*p* < 0.05) was an independent predictor of poor prognosis.

**Conclusion:** Non-invasive ultrasonic ONSD could be useful in improving the prognosis of patients with AIS receiving intensive care.

## 1 Introduction

Acute ischemic stroke (AIS) can lead to brain edema, accompanied by increased intracranial pressure (ICP), resulting in decreased cerebral perfusion pressure. At the same time, with an increase in ICP, brain hypoxia and secondary brain injury may occur, aggravating the patient’s condition. The patient may have symptoms such as headache, drowsiness, coma, and even brain hernia, which will have a negative impact on neurological prognosis (Seyedhosseini et al., 2019; Lee et al., 2020b). Monitoring changes in ICP over time is very important for the treatment and prognosis of patients. However, invasive monitoring in patients with high ICP is often considered extremely risky and challenging because of the risk of bleeding (7%) (Bauer et al., 2011) and infection (5–20%) (Beer et al., 2008). Invasive ICP monitoring also requires special equipment and neurosurgeons; therefore, it is not widely available. Lumbar puncture is a commonly used ICP evaluation method in the clinic. However, the procedure is associated with risks such as headaches, infections, and bleeding, making it unsuitable for compromised patients. Lumbar puncture may be contraindicated in emergent or critically ill patients.

These factors are responsible for many patients not being monitored by ICP in a timely manner. Therefore, for many years, the accepted, empirical approach to treating patients with high cranial pressure did not include knowledge of ICP measurements. Unfortunately, in the early stages of the disease, if the patient’s symptoms do not change significantly, symptoms are not clearly expressed or cannot be expressed, and elevated ICP cannot be found as soon as possible, then the treatment may be delayed. The use of computed tomography (CT) or magnetic resonance imaging (MRI) is needed to transport patients, which may cause secondary injury to critically ill patients. And compared with CT, MRI has a higher resolution, but requires more time to perform. In addition, if the medication time is too long after the use of ICP reduction treatment, excessive use of ICP reduction drugs may cause renal impairment (Moustafa et al., 2021), central pontine myelinolysis, and rebound internal hypertension exists (Qureshi and Suarez, 2000). Therefore, the development of non-invasive dynamic monitoring of ICP is of great significance for patients with AIS.

In recent years, ultrasonic measurement of optic nerve sheath diameter (ONSD) has attracted increasing attention as a new non-invasive repeatable method for estimating ICP (Zhang et al., 2017; Seyedhosseini et al., 2019). The optic nerve is ensheathed by three layers of meninges (the dura, arachnoid, and pia meninga), collectively known as the optic sheath. The space between the sheath is the continuation of the brain space, which is filled with cerebrospinal fluid and ICP changes are transmitted in the intracranial subarachnoid space (Wang et al., 2018; Lochner et al., 2019). In previous studies, the change in ONSD was highly consistent with that in ICP. It was suggested that the ultrasonic measurement of ONSD can dynamically evaluate ICP in real time.

In clinical practice, timely assessment of ICP can help patients and clinical workers make accurate and rapid treatment decisions. This may be particularly beneficial to patients who resenting to the emergency department due to elevated ICP. In addition, it may play an important role in the treatment of patients (Lee et al., 2020b). However, whether monitoring ICP using point-of-care ultrasound (POCU) for ONSD could improve the prognosis of AIS patients is not clear. This study aimed to investigate the clinical value of ONSD in AIS prognosis in patients receiving intensive care.

## 2 Materials and methods

### 2.1 Study setting and population

Patients were recruited from the First Hospital of Jilin University, a general public hospital in China. Patients from the neurological intensive care unit (NICU) were recruited between December 2019 and July 2021. This study was approved by the Ethics Committee of the First Hospital of Jilin University (approval number: 19K129-001). All patients or their relatives voluntarily participated and signed the informed consent forms.

Patients who met the following criteria were included in our study: patients who met the AIS diagnostic criteria in the “Chinese Acute Ischemic Stroke Diagnosis and Treatment Guide 2018,” who presented with stroke-related symptoms and were diagnosed with ischemic lesions by CT or MRI, and patients with anterior circulation infarction within 24 h from onset to admission. The exclusion criteria were as follows: (1) < 18 years of age; (2) transient ischemic attack; (3) any eye diseases, such as certain optic neuropathy, glaucoma, eye trauma, or tumor; (4) convulsing or restlessness and inability to cooperate with the examination; (5) head trauma, intracranial hemorrhage; (6) known pathologies such as intracranial mass or hydrocephalus; (7) metabolic disorder (e.g., uremia, hepatic failure, hypothyroidism, hyperthyroidism); (8) pregnancy; (9) scheduled surgical treatment, including intravascular interventional therapy; and (10) use of central nervous system depressants, such as sedatives.

**FIGURE 1:**
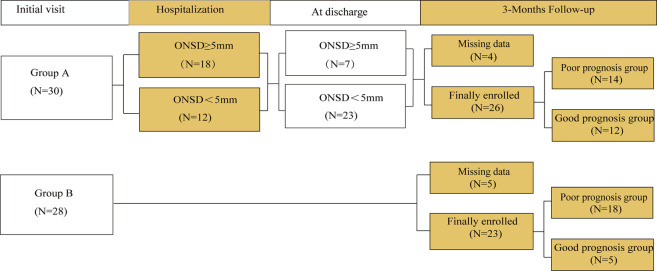
Groups setting and follow-up.

The patients were divided into groups A and B according to whether they or their relatives would accept patients for ONSD monitoring (Figure 1). In group A, ICP was monitored using ultrasonic ONSD once a day until the patients were discharged from the hospital. During the monitoring process, patients with ONSD ≥5 mm (Maissan et al., 2015) must be treated by clinicians to lower ICP. Patients in group B received routine clinical treatment in accordance with guidelines (Chinese Society of Neurology, 2018) by combining clinical symptoms and imaging examinations.

The baseline information collected from the patients was as follows: (1) demographic and physiological data including age, sex, body mass index (BMI), waist circumference, head circumference, systolic blood pressure (SBP), and diastolic blood pressure (DBP); (2) Past medical history: history of stroke, hypertension, diabetes, dyslipidemia, and smoking. (3) The time of onset and level of consciousness of patients using the Glasgow Coma Scale (GCS) score.

Dr. Zhang followed all patients either in the clinic or by telephone using the Glasgow Outcome Scale (GOS). All patients were followed up at discharge and once a week for 3 months. In the GOS score, scores of four to five were indicative of good prognosis, and scores of one to three were indicative of poor prognosis. During the follow-up period, the patient’s survival status was recorded, and the date of death was recorded if the patient died.

### 2.2 Examination

All patients were scanned with 1.5T brain CT/MRI at admission. These were examined by radiologists with 7 years of experience who knew nothing about the severity of the patient.

**FIGURE 2:**
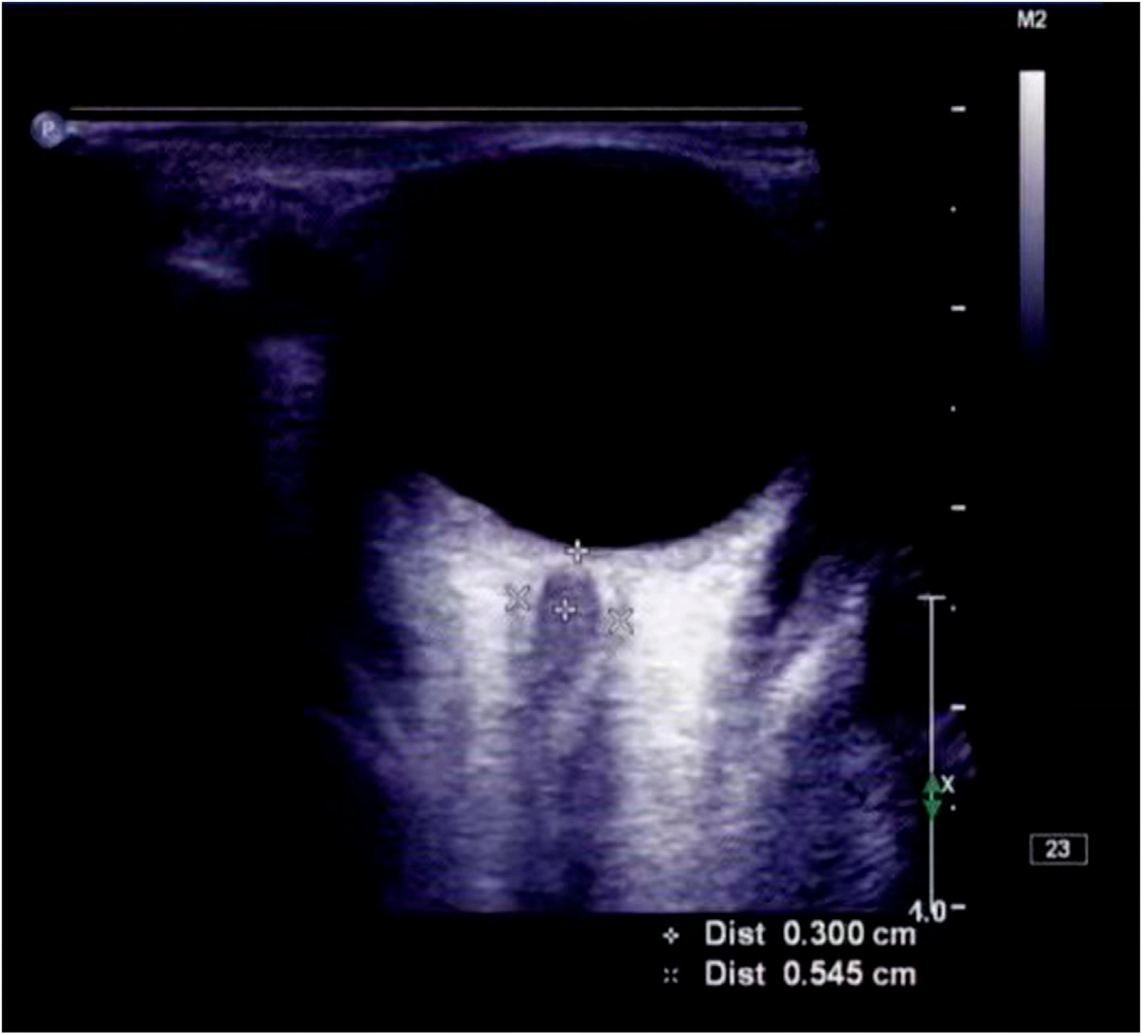
ONSD was measured by ultrasound. The ONSD, as measured with transorbital ultrasonography, was 0.545 cm, in this patient (a man in his early 60s) with acute ischemic stroke.

Ultrasonic ONSD examinations were performed in B-mode using a Philips iU22 ultrasound system (Andover, MA, United States) and 9- 3-MHz linear array transducer. The surgery was performed by two experienced doctors (J-L W, C L) who were unaware of each other’s results. Both doctors adjusted the acoustic output to meet the principle of “reasonable realization as low as possible.” (Toms, 2006). The patient lay on the bed, and a probe was placed gently on the closed upper eyelid with a thick layer of ultrasound gel. In line with previous protocols, the ONSD was assessed 3 mm posterior to the point where the optic nerve entered the globe (Figure 2). Two measurements were taken for each ONSD. One measurement was performed in the sagittal plane (with the probe vertical) and one was performed in the transverse plane, and the mean value of eight measurements was recorded.

### 2.3 Statistical analyses

The data were analyzed using SPSS software (version 25.0; United States). Continuous variables were represented as mean ± standard deviation (SD) or median with quartile interval (IQR), and categorical variables were expressed as frequency and percentage. Histograms and Kolmogorov-Smirnov tests were used for normal distributions. Within the group, the chi-square test was used for qualitative data, and the Mann-Whitney *U* test was used for quantitative data in single factor analysis. An independent samples *t*-test was used to compare the two groups for the analysis of quantitative data. Spearman correlation analysis was used to identify the relationships between qualitative data. The Kaplan-Meier method was used to draw a good prognosis rate curve, and the log-rank test was used to compare the difference in good prognosis rates between groups A and B. Finally, univariate and multivariate Cox proportional hazards regression analyses were used to identify possible predictors of adverse outcomes. Variables that were statistically significant in the univariate analysis were included in the multivariate analysis and the final Cox model was defined. All data were statistically significant at *p* < 0.05 (double tail).

## 3 Result

### 3.1 Demographic analysis

In total, 58 subjects (69% males) were included in the study. The following subjects were excluded: two cases of lost follow-up, one case of ocular trauma, four cases of intravascular intervention, and two cases of time-window defects. There were 49 patients (mean age, 65 ± 9 years; range, 44–85 years; 75.5% males) with complete anterior circulation syndrome (22 cases) and partial anterior circulation syndrome (27 cases). Group A included 26 patients (mean age, 65 ± 10 years; range, 44–85 years; 80.8% males), with a mean ONSD of 5.01 ± 0.48 mm (range, 4.03–5.90 mm) and a mean GCS score of 8 ± 3 (range, 3–13). Group B included 23 patients (mean age, 65 ± 8 years; range, 49–79 years; 69.6% males), with a mean GCS score of 7 ± 3 (range, 3–12). There were no significant differences at baseline between the two groups (Table 1).

**TABLE 1:** Baseline demographics and clinical characteristics.

	GROUP A (26)	GROUP B (23)	95%CI	P-Value
Sex(male)	21 (80.8%)	16 (69.6%)	-0.126–0.343	0.363
Age (years)	65 ± 10	65 ± 8	-5.182–5.527	0.949
GCS score	8 ± 3	7 ± 3	-0.450–2.524	0.167
BMI (kg/m2)	23.9 ± 4.6	24.7 ± 3.3	-3.076–1.592	0.525
Waistline (cm)	89.2 ± 15.9	85.7 ± 14.4	-5.265–12.268	0.426
Head circumference (cm)	55.8 ± 1.4	55.8 ± 1.3	-0.835–0.722	0.884
SBP (mmHg)	156 ± 11	157 ± 14	-8.513–5.821	0.707
DBP (mmHg)	86 ± 11	86 ± 7	-5.485–5.645	0.977
Massive cerebral infarction (Yes)	20 (76.9%)	16 (69.6%)	-0.167–0.311	0.560
Midline shift (Yes)	6 (23.1%)	10 (43.5%)	-0.057–0.435	0.129
Ventricular pressure (Yes)	10 (38.5%)	12 (52.2%)	-0.133–0.382	0.336
History of stroke (Yes)	6 (23.1%)	9 (39.1%)	-0.094–0.395	0.224
Atrial fibrillation (Yes)	3 (11.5%)	8 (34.8%)	-0.004–0.449	0.052
Hypertensive (Yes)	23 (88.5%)	21 (91.3%)	-0.168–0.214	1.000
Diabetes (Yes)	18 (69.2%)	14 (60.9%)	-0.173–0.330	0.539
Dyslipidemia(Yes)	24 (92.3%)	20 (87.0%)	-0.132–0.252	0.655
Smoke (Yes)	19 (73.1%)	16 (69.6%)	-0.207–0.279	0.786
Hospitalization days	17 ± 11	18 ± 11	-7.943–4.796	0.622

Abbreviations: SBP, systolic blood pressure; DBP, diastolic blood pressure; BMI, body mass index (calculated as weight in kilograms divided by height in meters squared); GCS, glasgow coma scale.

### 3.2 Analysis between operators

In group A, the Pearson correlation coefficients of the ONSD between the left and right eyes of the two observers were 0.937 and 0.916, respectively. Based on the Bland-Altman analysis, the average ONSD difference between the two observers was -0.009 (0.329) mm. The limits of agreement were 0.691 mm in the left eye and -0.683 mm in the right eye, respectively.

### 3.3 Correlation analysis at admission

ONSD was correlated with GCS score, with an r of -0.418 (*p* < 0.05). ONSD was correlated with midline shift, with an r of 0.584 (*p* < 0.05). The correlations between ONSD and the presence of ventricular pressure and massive cerebral infarction were 0.590 and 0.523, respectively (*p* < 0.05).

### 3.4 ONSD changes during hospitalization

During hospitalization, ICP was evaluated in group A evaluated ICP by ultrasonographic ONSD. Fifteen patients had ONSD ≥5.0 mm at admission, and nine patients had ONSD ≥5 mm after 1 week. At the time of discharge, seven patients had an ONSD ≥5 mm.

### 3.5 Results were compared between the two groups at discharge

At discharge, the GOS score was higher in group A than group B (3.35 ± 1.35 vs. 2.57 ± 1.121, *p* = 0.034, 95%CI, 0.061–1.501). The proportion of patients with a good prognosis was higher in group A than in group B (46.2% vs. 13.0%, *p* = 0.006). ONSD on admission was correlated with the GOS score, with an r of-0.648 (*p* < 0.05).

The Kaplan-Meier curve showed that there was no difference in the good prognosis rates between groups A and B (log-rank = 0.334).

### 3.6 Comparison of follow-up results and analysis of influencing factors

**FIGURE 3:**
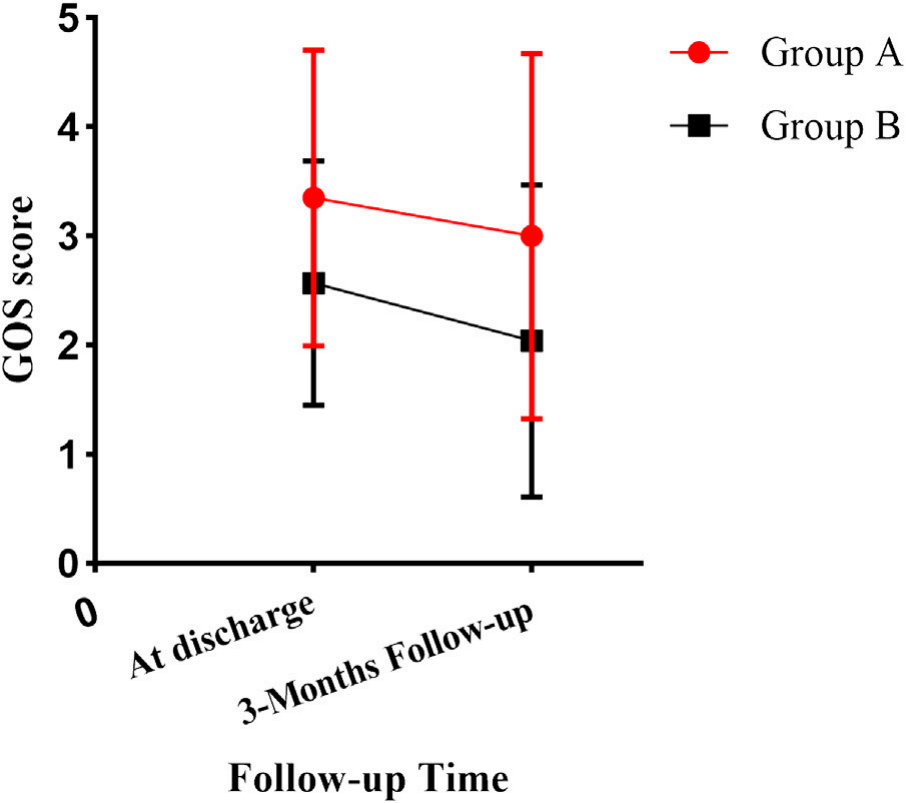
The differences in GOS scores between groups A and B were compared.

After 3 months of follow-up, the GOS score was higher in group A than in group B (3.00 ± 1.673 vs. 2.04 ± 1.430, *p* = 0.038, 95%CI, 0.056–1.857) (Figure 3). The proportion of patients with a good prognosis was higher in group A than in group B (46.2% vs. 21.2%, *p* = 0.039). ONSD on admission was correlated with the GOS score, with an r of -0.731 (*p* < 0.05).

**FIGURE 4:**
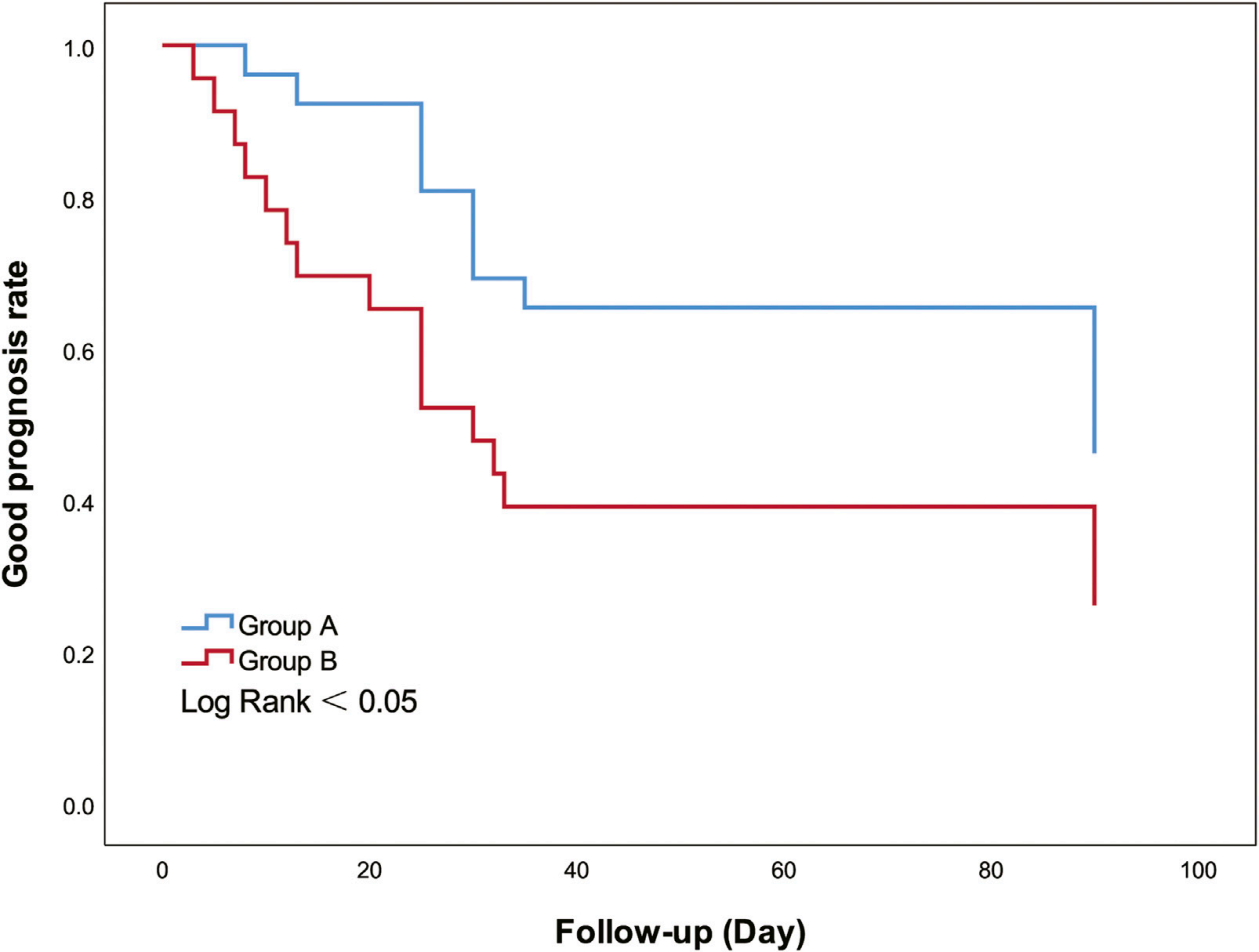
Kaplan-Meier curves show good prognosis rate between groups A and B. Group A had a higher good prognosis rate than group B (Log Rank = 0.045).

Kaplan-Meier curves were drawn (Figure 4), and univariate and multivariate Cox regression models were fitted (Table 2). The Kaplan-Meier curve showed that the good prognosis rate of group A was higher than that of group B (log-rank <0.05). In group A, to investigate potential confounding factors, univariate analysis was applied. Midline shift and ONSD were identified as statistically significant with poor prognosis. The main results (*p* < 0.05) of related parameters (midline shift and ONSD) were included in the multivariate analysis to obtain the final prediction model. In the multivariate Cox proportional hazard models, the adjusted hazard ratio of midline shift was 1.612 (95%CI, 0.453–5.730, *p* > 0.05), and that of ONSD was 4.77 (95%CI, 1.266–17.979, *p* < 0.05).

**TABLE 2:** Analysis of influencing factors.

	Univariate analysis	*p*-value	Multivariate analysis	*p*-value
	HR (95% CI)		HR (95% CI)	
Age (years)	1.038 (0.980–1.098)	0.203		
Sex (men)	0.337 (0.103–1.110)	0.074		
GCS score	0.834 (0.679–1.024)	0.084		
Atrial fibrillation (Yes)	1.421 (0.317–6.364)	0.646		
History of stroke (Yes)	0.854 (0.238–3.061)	0.808		
Ventricular pressure (Yes)	1.798 (0.630–5.131)	0.273		
Midline shift (Yes)	4.100 (1.403–12.008)	0.01	1.612 (0.453–5.730)	0.461
Massive cerebral infarction (Yes)	4.902 (0.641–37.503)	0.126		
ONSD (mm)	6.177 (1.988–19.187)	0.002	4.770 (1.266–17.979)	0.021
BMI (kg/m2)	0.984 (0.879–1.103)	0.786		
Waistline (cm)	0.992 (0.958–1.028)	0.666		
Head circumference (cm)	0.952 (0.658–1.379)	0.796		
SBP (mmHg)	0.975 (0.930–1.020)	0.304		
DBP (mmHg)	0.977 (0.932–1.023)	0.325		
Hypertensive (Yes)	0.771 (0.172–3.450)	0.734		
Diabetes (Yes)	0.663 (0.222–1.984)	0.463		
Dyslipidemia (Yes)	0.316 (0.065–1.528)	0.152		
Hospitalization days	0.964 (0.908–1.022)	0.219		
Smoke (Yes)	2.426 (0.543–10.839)	0.246		

Abbreviations: CI, confidence interval; HR, hazard ratio; SBP, systolic blood pressure; DBP, diastolic blood pressure; ONSD, optic nerve sheath diameter; BMI, body mass index (calculated as weight in kilograms divided by height in meters squared); GCS, glasgow coma scale.

## 4 Discussion

The overall prognosis of patients with AIS with ONSD monitoring in group A was better than that of patients in group B. Univariate and multivariate Cox proportional hazards models suggested that ONSD is an independent predictor of poor prognosis.

In this study, ONSD was found to be negatively correlated with GCS score by comparing the correlation between ONSD and GCS scores (*p* < 0.05). This result suggests that the ONSD is valuable for evaluating the condition of patients. With an increase in ONSD, the condition of the patients is poor. In addition, ONSD was positively correlated with massive cerebral infarction, midline shift, and ventricular pressure. This might indicate that increased ICP with increased stroke area or worsening stroke can be well demonstrated by ONSD. Similar results have been reported in previous studies. According to Gokcen et al.‘s stroke study (Gökcen et al., 2017), patients affected by complete anterior circulation infarction had the highest ONSD detection compared to other ischemic groups. The authors showed that ONSD can identify patients with middle cerebral artery stroke who are at high risk of developing malignant middle cerebral artery syndrome. In another study (Lochner et al., 2020), ONSD on the day of admission was associated with the assessment of cerebral infarction volume on CT scan on day 1 (r = 0.623; *p* = 0.002). It can be seen that monitoring ICP by ONSD is of great significance for the clinical application of critically ill patients. Thus, ONSD enables the early diagnosis of the patient’s condition.

Non-invasive ultrasound detection of ONSD can not only evaluate the condition of patients at admission but also has value in evaluating the prognosis of patients. ONSD at admission was significantly associated with GOS scores at discharge and at the 3-month follow-up. All variables were negatively correlated (*p* < 0.05). On univariate and multivariate Cox regression analyses, ONSD was found to be an independent predictor of poor prognosis. For every 1 mm increase in ONSD, the risk of poor prognosis increased by 3.77 times. These results suggest that ONSD has potential value for predicting patient prognosis. McKeown et al. (McKeown et al., 2022) concluded that the presence of a midline shift is a predictor of a poor prognosis. Similarly, in this study, ONSD was positively correlated with the midline shift (*p* < 0.05). This result suggests that ONSD may be a predictor of a poor prognosis.

This study showed that evaluation of ICP in AIS patients by monitoring ONSD may help improve the prognosis of patients. After randomization according to patients’ wishes, patients in group A were assessed for ICP changes by ONSD during hospitalization. There were statistically significant differences in the GOS scores at discharge and during follow-up between the two groups. The scores of the patients in group A were better than those in group B, indicating that the prognosis of the patients in group A was better than that in group B. In a comparison of good prognosis rates between the two groups, group A showed an advantage over group B. Yildiz et al. (Yildiz et al., 2021) demonstrated in clinical studies that rapid bedside ONSD measurements to accurately diagnose elevated ICP were superior to CT at an early stage. Patients with suspected ischemic stroke and elevated ICP were immediately initiated with successful results. In addition, in an article by Lee et al. (Lee et al., 2020a), it was proposed that an increase in the ONSD/eyeball transverse diameter ratio increases the chance of malignant progression of the disease and can be used as a marker for early urgent therapeutic intervention in patients with malignant middle cerebral artery infarction, which can reduce mortality and improve functional prognosis. Therefore, non-invasive bedside monitoring of ONSD in stroke patients is of great significance in improving patient prognosis.

The POCU monitoring of ONSD can quickly and dynamically evaluate changes in ICP within 5 min, which is suitable for patients with severe diseases. For critically ill patients who are seriously ill and subject to rapid changes in their condition, ONSD is very meaningful for real-time ICP evaluation. In a previous study (Chen et al., 2019), ONSD measurements were performed approximately 5 min before and after lumbar puncture (LP). With a decrease in cerebrospinal fluid pressure, ONSD decreased immediately in 95% of subjects. ΔONSD and ΔICP were significantly correlated (r = 0.451, *p* < 0.01). Similarly, in the Piergiorgio Lochner study (Lochner et al., 2020), four patients with malignant stroke also had a synchronous decline in ONSD after decompressive craniectomy, which indicates that ONSD has potential clinical application value in monitoring after decompressive craniectomy.

In addition, the POCU monitoring of ONSD has the advantage of price and bedside operation, which makes it more convenient for severely ill patients who are not suitable for transportation. ONSD is simple and easy to learn. Previous studies have shown that a short duration of ONSD training for residents can achieve good consistency (Shah et al., 2009; Bäuerle et al., 2013; Padayachy et al., 2016; Wang et al., 2017). The above characteristics of ONSD measured by ultrasound suggest that POCU of ONSD can be recommended as a new technique in the intensive care unit.

## 5 Limitations

This study had some limitations. This study was conducted at a single center, and a larger sample size is needed for further verification.

## 6 Conclusion

Non-invasive ultrasound measurement of ONSD could evaluate the condition of patients with AIS and may be beneficial to improve the prognosis of patients.

## Data Availability

The raw data supporting the conclusion of this article will be made available by the authors, without undue reservation.

## Abbreviations

AIS, acute ischemic stroke; BMI, body mass index (calculated as weight in kilograms divided by height in meters squared); CI, confidence interval; CT, computed tomography; DBP, diastolic blood pressure; HR, hazard ratio; ICP, intracranial pressure; GCS, Glasgow coma scale; GOS, Glasgow outcome scale; LP, lumbar puncture; MRI, magnetic resonance imaging; NICU, neurological intensive care unit; ONSD, optic nerve sheath diameter; POCU point-of-care ultrasound; SBP, systolic blood pressure.
